# Establishing an international laboratory network for neglected tropical diseases: Understanding existing capacity in five WHO regions

**DOI:** 10.12688/f1000research.16196.4

**Published:** 2018-12-02

**Authors:** Laura Dean, Janet Njelesani, Charles Mulamba, Russell Dacombe, Pamela S. Mbabazi, Imelda Bates

**Affiliations:** 1 Capacity Research Unit, Liverpool School of Tropical Medicine, Liverpool, UK; 2 Steinhardt School of Culture, Education, and Human Development, New York University, New York, USA; 3 Global Working Group on Capacity Strengthening for National NTD Programmes, World Health Organization, Geneva, Switzerland

**Keywords:** Neglected Tropical Diseases, Capacity Building, Laboratory Networks, Quality Assurance, Americas, Eastern Mediterranean, Europe, South-East Asia, Western Pacific

## Abstract

**Background.** Limited laboratory capacity is a significant bottleneck in meeting global targets for the control and elimination of neglected tropical diseases (NTD). Laboratories are essential for providing clinical data and monitoring data about the status and changes in NTD prevalence, and for detecting early drug resistance. Currently NTD laboratory networks are informal and specialist laboratory expertise is not well publicised, making it difficult to share global expertise and provide training, supervision, and quality assurance for NTD diagnosis and research. This study aimed to identify laboratories within five World Health Organisation regions (South-East Asia, Eastern Mediterranean, Americas, Western Pacific and Europe) that provide NTD services and could be regarded as national or regional reference laboratories, and to conduct a survey to document their networks and capacity to support NTD programmes.

**Methods.** Potential NTD reference laboratories were identified through systematic searches, snowball sampling and key informants.

**Results.** Thirty-two laboratories responded to the survey. The laboratories covered 17 different NTDs and their main regional and national roles were to provide technical support and training, research, test validation and standard setting. Two thirds of the laboratories were based in academic institutions and almost half had less than 11 staff. Although greater than 90 per cent of the laboratories had adequate technical skills to function as an NTD reference laboratory, almost all laboratories lacked systems for external verification that their results met international standards.

**Conclusions.** This study highlights that although  many laboratories believed they could act as a reference laboratory, only a few had all the characteristics required to fulfil this role as they fell short in the standard and quality assurance of laboratory processes. Networks of high quality laboratories are essential for the control and elimination of disease and this study presents a critical first step in the development of such networks for NTDs.

## Introduction

Laboratories are recognised as one of the weakest elements of health systems due to chronic under-investment. Lack of investment results in poor infrastructure, inadequate numbers and skills of technical staff, insufficient and uncoordinated technical assistance, and lack of diagnostic tools appropriate for low-resource settings ^1, 2^. Yet laboratory services are integral to interventions for the surveillance, control and elimination of neglected tropical diseases (NTDs). Laboratories provide clinical and monitoring data about disease prevalence and trends, and are essential for flagging up early signs of drug resistance ^1, 3– 6^. Appropriate management of clinical cases of NTDs depends on laboratories providing accurate diagnoses for identifying cases ^5, 6^. Preventive chemotherapy interventions through mass drug administration (MDA) rely on laboratory data to make decisions regarding intervention effectiveness and for reliably documenting progress towards zero transmission ^5^.

Accelerated scale-up of existing interventions is critical to reach the 2020 NTD Roadmap targets on the control and elimination of NTDs, however, lacking laboratory capacity is a critical bottleneck preventing the international NTD community from meeting targets. There needs to be enhanced laboratory ability in areas with significant NTD prevalence to provide technical and scientific support for the diagnosis, surveillance, monitoring and evaluation of national NTD programmes ^7^. The World Health Organisation’s (WHO) Strategic and Technical Advisory Group (STAG) for Neglected Tropical Diseases have therefore prioritised strengthening the capacity of NTD laboratories and establishing a formal NTD laboratory network which can provide a quality assurance and referral function ^8^. 

Globally, few laboratories specialise in NTDs. Laboratory support for NTD programmes is generally provided by parasitology laboratories within national health care systems or research institutions ^9^. Most of these laboratories focus on malaria, and to a lesser extent on soil transmitted helminths (STH), with very little laboratory expertise in other NTDs ^10^. No central register of specialist NTD laboratories exists. NTD laboratory expertise is fragmented and un-coordinated, with no formal referral system or network to provide high level support from internationally-accredited reference laboratories for quality assurance of NTD testing. Consequentially, much laboratory data available on NTDs, that has been used to make important strategic decisions about programme implementation and transmission rates, may have been generated by laboratories working in isolation that are not enrolled in any external quality assurance scheme. The global laboratory infrastructure for NTD control programmes lags behind many other global health programmes, such as those for tuberculosis, malaria, poliomyelitis, measles and hepatitis, which successfully established a globally connected network of laboratories and systems for externally validating disease-specific laboratory data as recommended by the World Health Assembly ^11, 12^.

To identify and harness existing capacity and to improve efficiency, laboratories that support national NTD programmes need to be mapped and organised into a functional international network. At the top tier, there should be internationally accredited and interlinked national reference laboratories. Each of which should head a pyramidal referral structure comprising laboratories at, for example, provincial level who support more peripheral district and primary care sites involved in front-line diagnosis and surveillance. A requirement of NTD reference laboratories is to maintain their own accreditation and service quality, and to facilitate provision of quality services by lower level laboratories through, for example, offering training on good laboratory practice and quality management systems, external quality assessment and referral testing, and monitoring performance standards through the organisation of regular proficiency testing ^1^.

Information about the location and expertise of laboratories with specialist NTD expertise across WHO regions is scarce and difficult to access. It is not generally known whether these laboratories meet international accreditation standards for their NTD tests or have the capacity, expertise, and networks that would enable them to operate as national or regional reference centres. Creating a database of laboratories that includes a description of what support they can provide for NTD programmes is an essential first step in the process of establishing an international and regional NTD laboratory network.

This study aimed to identify laboratories that provide NTD services and could be regarded as national or regional reference laboratories within WHO regions, and to document their capacity to support NTD programmes. It covered five of WHO’s six regions since the WHO Africa region office conducted its own complementary study and the results could not be collated due to differing study methods. Our study mapped the geographical distribution and networks of these laboratories, and collated information about the skills and services they provided to support NTD programmes. Scoping the current situation provides a platform on which to design strategies to build an international network of accredited NTD reference laboratories. Such a network is essential to overcome the laboratory bottleneck which is a key barrier in accelerating intervention scale-up to meet 2020 NTD Roadmap goals ^7^.

## Methods

There is no existing global register of specialist NTD laboratories. This scoping study developed an unbiased and comprehensive way of identifying potential NTD reference laboratories in the five WHO regions – Americas, Eastern Mediterranean, Europe, South-East Asia and Western Pacific. As there was no pre-existing definition of an ‘NTD reference laboratory’, we extracted information from published literature ^13– 17^ about the laboratory characteristics needed to fulfil diagnostic, research, supervision, training, quality, and networking requirements of a national or regional reference laboratory for NTDs, and verified them with NTD control programme specialists. These characteristics were:

•  able to conduct verifiable quality diagnosis in one or more NTDs

•  able to support research into NTDs prevalent in their region

•  able to train and mentor staff in national or tertiary level laboratories within the region

•  actively networked with other national and international NTD laboratories and research institutions

•  evidence of accreditation of NTD tests to international standards (e.g., ISO 15189, Good Clinical Laboratory Practice (GCLP))

Detailed information from the literature about each of these characteristics was used to design an electronic survey administered through Bristol On-line Survey (now Online surveys) to laboratories in the five WHO regions with potential to be national or regional NTD reference laboratories. Topics covered were: location and geographical coverage, NTD tests available, accreditation status, staffing, ability to provide training and technical support, and any capacity gaps the laboratory perceived they had in relation to NTDs.

To identify as many potential regional reference laboratories to include in the survey, and to avoid bias, two wide-ranging search strategies were used. Firstly, key informants were identified from international NTD programmes and research institutions and through WHO regional offices. These included WHO officers in each of the five regions, representatives of multi-lateral agencies supporting laboratory networks and centres, and NTD funders and researchers. Snowballing was used to identify further key informants.

Each key informant was asked to identify which laboratories they were aware of that could be considered an NTD reference laboratory based on our pre-defined list of characteristics. Laboratories did not have to focus exclusively on NTDs, since NTDs may be part of a larger portfolio of work but needed to have a reputation as a referral laboratory (or laboratory unit) for NTDs. 25 key informants provided contact details for 69 laboratories that they considered may be perceived as an NTD reference laboratory.

Secondly, an internet search for potential NTD reference laboratories was conducted. Countries affected by NTDs in each of the five WHO regions were identified from information on NTD strategies and/or activities in documents on WHO regional websites ^18– 23^, from country-specific information in the WHO NTD roadmap, and from individuals in the WHO Global Working Group on Capacity Strengthening for national NTD programmes. Overall 60 countries were identified as being affected by and prioritising NTDs in the five WHO regions: The Americas 17 countries, South-East Asia 11 countries, Europe 8 countries, Eastern Mediterranean 14 countries, and Western Pacific 10 countries. Potential NTD reference laboratories were identified by searching websites of national NTD programmes in NTD-affected countries, and the websites of the WHO regional offices, and by following additional links and references provided on these websites. The internet search strategy identified 98 laboratories that may potentially be NTD reference laboratories.

For each identified laboratory, contact details of laboratory heads were obtained from the websites or through key informants. Overall the combined searches yielded 167 contacts in potential reference laboratories. Each contact person was provided with information about the purpose and content of the survey by e-mail and asked to complete the survey. In order to increase response rates, the Modified Dillman approach ^24^ was used which involved fortnightly reminders about the survey for a period of five weeks between October 2013-January 2014 until the survey closed. The survey was also offered in Spanish as appropriate. Following closure of the surveys, 35 telephone calls were made to collect information from non-respondents. These focussed particularly on the European and Americas regions due to low response rates and generated one additional completed survey.

Data from the survey was entered into an excel spreadsheet and anonymised. Data was analysed to provide quantitative, descriptive information, and content analysis was conducted to identify NTD laboratories that met or were close to meeting the characteristics of a reference laboratory. 

## Results

### Response rates and geographical coverage

Nineteen percent (n=32) of the 167 of the laboratory heads contacted responded to the survey. The majority of respondents were from the Eastern Mediterranean region (34%, n=11) ( Table 1). No responses were received from the European region. The majority of laboratories (53%, n=17) provided a national level service and 25% (n=8) operated at the international level, predominantly within their own WHO region. The main regional role of surveyed laboratories was the provision of technical support (22%, n=7) and training (22%, n=7) to other laboratories though some were also involved in research, test validation and standard setting ( Table 1).

**Table 1.** Characteristics and distribution of potential neglected tropical disease (NTD) reference laboratories.

Main characteristics	Laboratories n=32 (%)
Laboratory location (by WHO region)	
Eastern Mediterranean	11 (34)
South-East Asia	10 (31)
Western Pacific	8 (25)
The Americas	3 (9)
Europe	0 (0)
Geographical coverage	
National	17 (53)
International	8 (25)
Sub-national	7 (22)
Regional Role	
Technical support to other laboratories	7 (22)
Providing training for other laboratories	7 (22)
Developing, validating and testing NTD methods and protocols	5 (16)
Advising on, and meeting, NTD research needs	5 (16)
Taking part in standardization on accreditation and certification	2 (6)
Other	6 (19)
Total number of staff in the laboratory *	
1–10	15 (47)
11–30	9 (29)
31–50	3 (9)
More than 50	5 (16)
Self-identified capacity gaps	
Lack of external Quality Assurance	9 (28)
Insufficient capacity to provide technical support	4 (13)
Insufficient expertise to conduct quality control of laboratory activities linked to NTDs	3 (9)
Insufficient capacity to conduct NTD diagnostics	3 (9)
Insufficient capacity to conduct NTD research	3 (9)
Insufficient capacity to support staff training	2 (6)
Inability to promote strategic plan	1 (3)
Insufficient funds	1 (3)
Insufficient human resource	1 (3)
None	5 (16)

*staff were not necessarily working full-time on NTD tests

### Resources and capacities

Laboratories tended to be small with almost half (47 %, n=15) employing 1–10 staff and three-quarters (76%) employing 30 or less. The South-East Asia region had the highest number of staff per laboratory. The Eastern Mediterranean region had the least staff and commonly lacked quality officers, management and administrative staff. Ninety one percent (n=29) of laboratories indicated that their staff had the necessary technical skills to function as an NTD reference laboratory and only one laboratory strongly believed it lacked this technical capacity. Two thirds (69%, n=22) of the laboratories were based in academic institutions and felt they were strong in supporting NTD research. The majority of laboratories (78 %, n=25) identified at least one gap in their capacity, most commonly external quality assurance, which was reported as lacking by 9 (28%) laboratories. The type of capacity gaps was similar across all five regions.

### NTD specialisation

The 32 laboratories covered 17 different NTDs across five WHO regions with 23 laboratories (72%) covering two or more NTDs. Most laboratories within the Eastern Mediterranean region (73%, n=8) specialised in leishmaniasis. Within the Western Pacific region, laboratories tended to focus on STH (50%, n=4) and schistosomiasis (50%, n=4). Laboratories in South-East Asia focused on STH (60%, n=6) and lymphatic filariasis (60%, n=6). Chagas disease, taeniasis, cysticercosis, echinococcosis, onchocerciasis and dengue were the only NTDs covered in the Americas region.

### International standards, quality assurance for NTD testing and networks

There was variation in adherence to laboratory quality standards. Only four (13%) stated adherence to international standards such as Good Laboratory Practice, ISO 15189 and ISO 9000. 47 percent (15) of laboratories adhered to national quality standards and 40% (13) did not adhere to any. Although 47% (19) of laboratories reported a quality officer, only five (16%) participated in an external quality assurance (EQA) programme for NTD tests. Four of these were in the Eastern Mediterranean region. All five stated that less than 5% of their results within the last 3 years had been unsatisfactory. In the Western Pacific, South-East Asia and Americas regions, at least 90% (12) of laboratories did not participate in an external quality assurance scheme. Fourteen (44%) laboratories had regular interactions with international NTD networks including Drugs for Neglected Diseases Initiative, Tropical Disease Research and regional NTD elimination programmes. International conferences, regional meetings, and NTD workshops, were the predominant modes of networking reported. Despite the majority (91%) of laboratories believing they have the capacity to carry out the role of a reference laboratory, only 14% (n=3) met all the pre-determined characteristics.

De-identified survey data Click here for additional data file. Copyright: © 2018 Dean L et al. 2018 Data associated with the article are available under the terms of the Creative Commons Zero "No rights reserved" data waiver (CC0 1.0 Public domain dedication).

## Conclusions

This study marks an important step in a process towards creating the need and awareness for an international network of NTD expert laboratories. We used a systematic and wide-ranging approach to identify 32 laboratories distributed across four of five WHO regions which have potential to be regional or national reference laboratories for NTDs. Between them, these laboratories reported that they have the technical skills to provide expertise in 17 different NTDs with each laboratory focussing on NTDs that are prevalent in their region. These laboratories could form the top tier of an interlinked network of laboratories capable of providing quality information for NTD programmes and able to act as NTD research and training centres. Half the laboratories operated at national level and a quarter at regional level and this two-level geographical focus forms a sound basis for creating national and international NTD laboratory networks.

Although over 90% of the laboratories surveyed in this study believed they could act as a reference laboratory, only 3 had all the characteristics required to fulfil this role. Almost all laboratories fell short in the area of standards and quality assurance of laboratory processes. A consistent finding was that 87% of laboratories did not adhere to international quality standards and 40% did not adhere to national quality standards. Further evidence of the paucity of quality systems is that only five of the 32 laboratories participated in an EQA programme for NTD tests; four of these were in the Eastern Mediterranean region. This means there is no independent verification that laboratory results meet international standards. Such verification is imperative in order to generate reliable results and lack of verification undermines confidence in data concerning NTD prevalence, trends and reduced drug efficacy ^25^. Unless NTD data originates from quality assured, accredited laboratories, reports about progress towards global NTD targets will lack credibility.

It is not clear why laboratories felt that they were able to act as reference laboratories even though many did not adhere to national or international quality standards and most were not enrolled in external quality schemes. This finding suggests that the importance of being able to demonstrate that test results are reliable may be under-recognised even among laboratory professionals and that this validation is not demanded by NTD programme managers and other decision makers.

### Limitations

Overall, we contacted 167 laboratories and received information from 32, a third of which were located within the Eastern Mediterranean region; none were in the European region. We used a broad search strategy, so a high response rate was not anticipated since it was likely that many laboratories contacted were not involved in reference-level NTD work. However, it is possible that our search missed some relevant laboratories or that we did not identify some laboratories because contact details were incorrect, or language barriers prevented some managers from responding. The methods used to identify laboratories may have biased the results towards those that are well-known or well-publicised and self-reporting could have led to over-estimation of the capacity of laboratories. The willingness of these laboratories to act as regional reference laboratories was not assessed as part of this study.

### Recommendations

The majority of laboratories covered at least two different NTDs. This diversity raises an important question about whether each laboratory should focus on one NTD or several. This will in part be dictated by existing expertise and by the burden of different NTDs in the vicinity. The most efficient use of resources may be to centralise expertise for several NTDs within one laboratory. This would facilitate throughput of large numbers of samples and centralise the expensive, state-of-the-art diagnostic tools needed to provide high levels of diagnostic specificity and sensitivity ^26– 28^. Amalgamation of laboratory services for several NTDs is complex and would need to be carefully managed to maintain rigorous systems and quality standards across a large range of services and to get buy-in from national programme managers and other laboratories ^25^.

Most of the laboratories specialising in NTDs are based in research institutions. This is an important factor to consider when planning an international NTD laboratory network since the primary goal of these laboratories is to generate research. This goal may not always be aligned with the priorities of national NTD programmes to provide routine service delivery and training. Research laboratories are characterised by short-term projects with high staff turnover and are strongly influenced by the topical interests of donors. The difference in priorities faced by research laboratories and NTD programmes means that potential tensions need to be anticipated and managed if a research laboratory is the primary provider of national or regional NTD laboratory expertise.

Although only three of the identified laboratories had all the characteristics of a reference laboratory, within each of the four WHO regions surveyed there are at least two laboratories that could be strengthened to reach international accreditation status for NTD tests and fulfil a role as a regional reference centre for NTDs. Responses from these laboratories indicated they have some experience in implementing international laboratory standards, in using advanced diagnostic tools and in providing technical support and training to other laboratories. At least some of the surveyed laboratories have the potential to be in the top global tier as regional reference laboratories once they are able to adhere to international quality standards. If they are integrated into a formal laboratory network, they will be able to, for example, share equipment, develop standardised indicators and regular monitoring protocols for laboratory performance and staff competence ^29^, and provide training, supervision and mentoring for lower tier laboratories.

This study provides preliminary information about the location and expertise of high-level laboratories specialising in NTDs. The data we collected was self-reported by laboratory personnel, so an important next step will be for additional information from European and African NTD laboratories to be incorporated, and for selected laboratories to undergo a more in-depth and independent assessment of their capacity. Criteria then need to be agreed and used to strategically select a small number of laboratories in each region which will be supported technically and financially to achieve international accreditation for NTD tests and formal recognition as regional NTD reference laboratories. These laboratories can then form foci around which to construct a global network of NTD laboratories with the longer term aim of encompassing national and sub-national laboratories within the network. A wide-ranging stakeholder consultation process, likely conducted under the stewardship of the NTD STAG which reports directly to the Director General of WHO, will be needed to define the criteria for selecting laboratories and to define the goal and operation of the NTD laboratory network ^30^. This should be supplemented by in-depth studies including interviews with laboratory staff and users, and decision-makers, to understand the requirements and context in which such laboratories need to operate. Only when this laboratory network is operational will it be possible to have effective and rapid regional and global referral and quality assurance systems and to have confidence in the NTD test results that are essential to support NTD programme operations and research needs ^12, 27^.

## Data availability

The data referenced by this article are under copyright with the following copyright statement: Copyright: © 2018 Dean L et al.

Data associated with the article are available under the terms of the Creative Commons Zero "No rights reserved" data waiver (CC0 1.0 Public domain dedication). 

Dataset 1: De-identified survey data 10.5256/f1000research.16196.d217501 ^31^

## Consent

Potential participants were informed about the purpose and content of the survey and could choose whether or not to complete the survey.

## Supplementary material

Supplementary File 1: World Health Organization (WHO) regions survey. Includes questions and possible responses
