# Supplementary material for: Establishing an international laboratory network for neglected tropical diseases: Understanding existing capacity in five WHO regions
Source: F1000Res. 2018 Dec 2;7:1464. Originally published 2018 Sep 14. [Version 4] doi: 10.12688/f1000research.16196.4 (PMC6509956; doi:10.12688/f1000research.16196.4)
Supplement: Supplementary file 2 [file f1000research-7-18921-s0001.tgz › aabfd21a-825a-4ff7-a29b-f76a0c6b951b_WHO_Regions_Survey.pdf]

WHO Regions Survey

Q	Statement	Response Scale
1	Is your laboratory considered to be a Regional Reference Laboratory for NTDs (defined as a centre/laboratory unit that offers technical and scientific support in the field of at least one NTD)? The following diseases are recognized as NTDs by WHO: • Buruli Ulcer • Chagas disease • Dengue • Dracunculiasis • Echinococcosis • Foodborne trematodiases • Human African trypanosomiasis • Leishmaniasis • Leprosy • Lymphatic filariasis • Onchocerciasis • Rabies • Schistosomiasis • Soil transmitted helminthiases • Taeniasis/Cysticercosis • Trachoma • Yaws	Yes<br>No
1a	Is your laboratory a RRL that does not specialise in NTDs?	Yes<br>No
1b	Is your laboratory a NTD speciality laboratory?	Yes<br>No
2	Are you	Male<br>Female
3	What is your age?	Under 25<br>26-35<br>36-45<br>46-55<br>56-65<br>Over 65
4	Which of the following best describes your work role?	Consultant<br>Director<br>Government employee<br>Lecturer<br>Manager<br>Research<br>Other
5	May WHO/LSTM have your permission to share your information in a global map/list of RRLs for NTDs?	Yes<br>No
6	May we contact you if more information is needed?	Yes<br>No
6a	Please provide an email address that we are able to contact you on.	Open free text space
7	Please indicate the official name of your laboratory	Open free text space
8	Please indicate the address of your laboratory	Open free text space
9	Please indicate the contact details of your laboratory (e.g., email, website, phone number)	Open free text space
10	What is the operational status of your laboratory?	Operational<br>Closed<br>Under construction
11	What is the ownership of your laboratory?	Government<br>Academic institute<br>Private<br>Other (open free text space)
12	Which NTDs are covered by your laboratory?	Buruli Ulcer<br>Chagas disease<br>Dengue<br>Dracunculiasis<br>Echinococcosis

		Foodborne trematodiases<br>Human African trypanosomiasis<br>Leishmaniasis<br>Leprosy<br>Lymphatic filariasis<br>Onchocerciasis<br>Rabies<br>Schistosomiasis<br>Soil transmitted helminthiases<br>Taeniasis/Cysticercosis<br>Trachoma<br>Yaws<br>Other (open free text space)
13	Which services does your laboratory offer?	Research<br>Diagnostics<br>Training<br>Technical support<br>Quality assurance<br>Quality control<br>Other (open free text space)
14	What geographical scope does your laboratory cover?	Sub-national<br>National<br>Regional (please specify the participating countries)
14a	If international, please specify the participating countries.	Open free text space
15	Does your laboratory have a strategic plan in place?	Yes<br>No
16	What quality standards (e.g., 5S, GCLP, ISO 15189) does your laboratory adhere to?	National standards/requirements/guidelines<br>International standards<br>Other (open free text space)<br>No specified standards
17	Please specify which quality standards apply to your laboratory.	Open free text space
18	Does your laboratory have a nominated quality control officer?	Yes<br>No
19	Does your laboratory have a nominated safety officer?	Yes<br>No
20	Does your laboratory participate in an external QA scheme?	Yes<br>No
20a	'''If yes''', please describe the external QA scheme.	Open free text space
20b	'''If yes''', which tests are sent for external QA?	Open free text space
20c	'''If yes''', what percentage of results in the external QA scheme in the last 3 years have been unsatisfactory?	Open free text space
21	Is there a national laboratory accreditation system in your country?	Yes<br>No
21a	'''If yes''', has your laboratory been accredited?	Open free text space
21b	'''If yes''', when did your laboratory receive this accreditation?	Less than 1 year ago<br>1-4 years ago<br>5-9 years ago<br>10-15 years ago<br>Over 15 years ago
22	Is your laboratory responsible for the management of an external quality assurance scheme?	Yes<br>No

22a	To which types of laboratories does your laboratory offer services?	Public<br>Private<br>Both public and private
22b	Which countries are the laboratories that your laboratory offers services to in?	Open free text space
22c	Is the participation of these laboratories in your external quality assurance scheme mandatory?	Yes<br>No
22d	To how many laboratories does your laboratory offer services?	0-10<br>11-50<br>51-100<br>101-500<br>> 500
22e	Do these laboratories pay a fee for your services?	Yes<br>No
22ei	If no, who covers the cost?	Open free text space
22f	How many times per year does your laboratory send out QA samples?	Open free text space
23	What is your laboratory's role regionally (region is defined as group of countries across international borders not regions within one country)?	Providing technical support to laboratories developing, validating and testing NTD methods and protocols<br>Providing QA/QC to laboratories<br>Providing training to laboratories<br>Encouraging laboratories to take part in inter-laboratory tests available at the national or regional level<br>Taking part in standardisation accreditation and certification<br>Advising on NTD research needs<br>Other (open free text space)
24	Does your laboratory interact or belong to any international NTD networks or organizations?	Yes<br>No
24a	'''If yes''', how many?	1<br>2-4<br>5-10<br>More than 10
24b	'''If yes''', please indicate which networks and organisations.	Open free text space
24c	'''If yes''', when was the last time your laboratory participated in an international network/organization event?	In the last week<br>In the last month<br>Within the last 6 months<br>More than 1 year ago<br>Never
24ci	Can you describe the last international network/organization event your laboratory participated in?	Open free text space
25	Does your laboratory carry out any training programmes for NTDs?	Yes<br>No
25a	What type of training is offered?	Research<br>Quality assurance<br>Quality control<br>Safety<br>Technical skills<br>Other (open free text space)
25b	Where is the training offered?	At your location<br>Outside of your location (e.g., onsite of another laboratory in your region)

		Other (open free text space)
25c	For whom is the training offered?	Directors of institutes<br>Laboratory managers<br>Laboratory scientists<br>Research officers<br>Quality control officers<br>Safety officers<br>Other (open free text space)
25d	How is the training funded?	Institutional funds<br>National competitive funding<br>International funding<br>Other (Open free text space
26	What types of research activities are undertaken at your laboratory?	Operational mapping of NTDs<br>Diagnostic tests<br>Impact of control programmes<br>Epidemiology and transmission of NTDs<br>Clinical management of NTDs<br>Other (open free text space)
26a	How is the research funded?	Institutional funds<br>National competitive funding<br>International funding<br>Other (Open free text space)
27	How many staff in total are employed in your laboratory?	1-10<br>11-30<br>31-50<br>More than 50
27a	Please tell us about any staff who are employed at your laboratory	
27ai	Number of lab scientists	Open free text space
27aii	Number of lab assistants	Open free text space
27aiii	Number of PhD students	Open free text space
27aiv	Number of post-doctoral researchers	Open free text space
27av	Number of research assistants	Open free text space
27avi	Number of administrative staff	Open free text space
27avii	Other	Open free text space
27b	How are the research staff positions funded?	Government funding<br>National competitive funding<br>International funding<br>Other (Open free text space)
27c	How are service staff positions funded?	Open free text space
28	To what extent do you agree with the following statement: ''my laboratory has the necessary human resources to carry out a role as a RRL''	Strongly disagree<br>Disagree<br>Agree<br>Strongly agree
29	To what extent do you agree with the following statement: ''my laboratory has the necessary technical capacities (e.g. supplies, equipment) to carry out a role as a RRL''	Strongly disagree<br>Disagree<br>Agree<br>Strongly agree
30	Which activity do you see as a capacity gap in your laboratory?	None<br>Research<br>Diagnostics<br>Training<br>Technical support<br>Quality assurance<br>Quality control<br>Other (open free text space

31	How are NTD services your laboratory offers funded?	Government funding<br>Research grants<br>National competitive funding<br>International funding<br>User fees<br>Other (Open free text space)
32	To what extent do you agree with the following: ''my laboratory has the necessary funding to carry out a role as a RRL.''	Strongly disagree<br>Disagree<br>Agree<br>Strongly agree
33	Which of the following techniques are undertaken by your laboratory?	Centrifuge<br>LED or fluorescent microscope<br>PCR<br>RT-PCR
33a	*If microscopy has been selected, please specify type of microscopy below. (e.g. light, fluorescent, phase contrast, electron, etc)	Open free space text
