# Supplementary material for: Establishing an international laboratory network for neglected tropical diseases: Understanding existing capacity in five WHO regions
Source: F1000Res. 2018 Dec 2;7:1464. Originally published 2018 Sep 14. [Version 4] doi: 10.12688/f1000research.16196.4 (PMC6509956; doi:10.12688/f1000research.16196.4)
Supplement: Peer review history

# Peer review: Reviewer response for version 4

**DOI:** 10.5256/f1000research.18921.r38348

**Published:** 2019-05-09

**Authors:** Christina Zarowsky

**Affiliations:** 1 Chronic Diseases Initiative for Africa (CDIA), Cape Town, South Africa; 2 École de santé publique, Université de Montréal, Montréal, Canada

This is a sound and useful contribution to the identified problem of improving laboratory capacity as a key dimension of meeting global NTD control targets. The structure, methods, findings, conclusions and recommendations are clear. The responses to previous reviewers' comments are adequate, both in the text and especially in the exchanges/comments directly. The content of the manuscript stands on its own as a useful though limited contribution. However, I found the gaps and limitations at least as intriguing and important and would encourage the authors  to comment a bit further on these (as in the exchanges with other reviewers), notably regarding the Africa region, the zero response from the European region, and what the implications might be of basing policy and funding decisions on the basis of evidence from this smallish sample (though on the pragmatic side, if the objective is to engage with laboratories and build a network, then there is a clear logic to engaging with the ones who responded to the survey).

 I wonder also if the authors might consider adjusting the title of the manuscript. The manuscript addresses some important dimensions of understanding existing capacity, though more in the form of documenting than analysing or understanding it. But the manuscript does not really address the main title, "Establishing an international laboratory network for neglected tropical diseases". The manuscript does not convincingly show that a NETWORK is the best or an appropriate response to the current situation (as opposed to strengthening the important gaps identified by the studies within countries or institutions, for example), nor does it engage at all with the dynamics, opportunities and challenges of creating and sustaining and mobilizing networks. I do not think that the study design and content lend themselves easily to this dimension, so I would suggest that the authors clarify somewhere up front or towards the end that the findings reported here may contribute to the eventual establishment of a network, and to outline up front at least briefly how they understand networks and why (a) network may be appropriate. If they prefer to refer readers to the NTD STAG recommendations, that is fine, but the paper on its own does not make a case for or directly contribute to the process of establishing a network.

 The other question that came to mind as I read the paper was how NTD reference (and other) laboratories are situated in the broader ecosystem of laboratories with disease control or public health functions in general. Another reviewer also commented on this parenthetically, but I would be interested in knowing a bit more. Is it the authors' and others' view that separate laboratories are needed (a vertical approach), or that NTDs should be integrated into other health system laboratory services (a fully integrated approach), or something in between (a diagonal approach)? Coming from the public health side of HIV, MNCH and NCD control, I am less than thrilled to envisage a parallel proliferation of separate, vertical structures on the laboratory side to what we see on the health system side. I would appreciate seeing a bit more on this, as I am a novice to the NTD laboratory world as an important domain in its own right.

 There are a few typo/grammatical awkwardnesses - paragraph 2, "lacking laboratory capacity" feels a bit awkward;  paragraph 4, incomplete sentence ("each of which should head a pyramidal..."), under methods I would not usually say that a study can "avoid bias" but rather "decrease, limit, etc".

I have read this submission. I believe that I have an appropriate level of expertise to confirm that it is of an acceptable scientific standard.

# Peer review: Reviewer response for version 3

**DOI:** 10.5256/f1000research.18633.r40768

**Published:** 2018-11-22

**Authors:** Linda Oskam

**Affiliations:** 1 DATOS B.V., Hoofddorp, The Netherlands

*My remarks are in italics. A major shortcoming at the moment is that it was impossible for me to track if and where changes have been made and I have very limited time to review as I am on the verge of traveling.*

*If there is no clear indication in the authors' response that something has been changed I had to assume that no changes were made. If changes were made after all, please indicate where and how text was changed as is common practice for revised articles.*

**

*Points that were not statisfactory addressed after this round of changes are: 1,3,4,5,8,10,11 and 12*

 1. The fact that data from the European and African regions are lacking is a major weakness of the article. I would suggest that the authors try to include at least the relevant laboratories from the African region.

**This work was undertaken by a separate group in the WHO AFRO region who have not agreed to share the results with us. We hope these results will be published by them.**

*Please indicate this more clearly in the text and also flag it as a weakness. In the article you state that it is due to a difference in methodology. If that is not the case, please indicate so.*

 2. The authors use a list of 25 NTDs. Which NTDs these are is unclear. WHO uses a list of 20 NTDs ( https://www.who.int/neglected_diseases/diseases/en/). It would be good to indicate which list of NTDs the authors use.

**We have changed the number in the article to 20 as per WHO list.**

*OK*

 3. Also: NTDs are a heterogeneous lot: parasites, viruses, bacteria, fungi. How realistic is it to assume that dedicated NTD laboratories exist? In most cases it will be one or more departments or groups within bigger laboratories.

**The intention was to included laboratories that dealt with one or some of the NTDs listed rather than all. The information gathered from this exercise was to be used to form a network of these heterogeneous labs.**

*And what is the advantage of that? Unclear to me.*

 4. With regard to accreditation: the laboratories that indicate that they have international accreditation: is the NTD testing portfolio included in their scope of accreditation? Otherwise it does not count as accredited lab.

**The NTD portfolio is included in their scope of accreditation.**

*Please indicate this in the text.*

 5. With regard to human resources: are the numbers in the table the total number of staff in the whole lab or the number of staff actually working on NTDs? The latter is the relevant number.

**This data was not recorded.**

*Please indicate in the text  as it is a limitation*

 6. Do the authors think that by identifying NTD labs through published literature they may have created a bias towards research-oriented laboratories? Many routine laboratory hardly publish their work (if at all).

**We did not only use published literature to identify laboratories but also used individuals from the WHO working group on Capacity Strengthening for NTD programmes who have a good knowledge of laboratories working on NTDs. We also used grey literature from targeted internet searches, for example searching websites of national NTD programmes. We also identified and interviewed key informants from international NTD programmes and WHO regional offices.**

*OK*

 7. Introduction, paragraph 4: “The role of the NTD reference laboratories is to maintain their own accreditation…” That is not a role, but a requirement to fully function as a reference laboratory. Supporting others to implement a QMS is a role.

**We have changed the wording in the article to make this clearer (i.e. from role to function).**

*OK*

 8. Methodology paragraph 5: “Overall 60 countries were identified as being affected by and prioritizing NTDs…” The WHO website (see reference under point 2) puts this number at 149. Please explain the discrepancy.

**This is due to not including WHO AFRO region.**

*149-60 = 89 and the African Region has only 55 countries according to the African Union. Please explain the difference*

 9. EQA programmes: I am not surprised that not many labs participate in EQA programs as I know from experience that these are often unavailable outside the big diseases. The ISO standard provides a loophole for this in the form of interlaboratory comparisons. Did the authors identify how many labs work with interlaboratory comparisons for their NTD testing?

**We did not collect this information.**

*OK*

 10. How many of the labs have direct reporting for NTDs to the national disease control programs at MoH level?  

**We did not collect this information.**

*Please indicate this somewhere in the text*

**

 11. I am afraid that a network of NTD laboratories is necessary, but will be very difficult to attain: most LMIC have a severe lack of lab staff and a severe lack of funding for their health system in general. NTDs will not be among their first 100 priorities. In many cases NTD testing will have to piggyback on other testing and make use of the leftover resources of the “big” diseases.

 Also, funding for NTD research is limited and haphazard. I think building a sustainable network will be an enormous challenge.

**We agree that the challenges to forming laboratory networks are large and hope this article will add to the debate.**

*Where is this indicated in the text?*

 12. I think that ultimately more qualitative research with multiple stakeholders (semi-structured interviews, focus group discussions) are needed to really understand how to best design and implement a sustainable laboratory network for NTDs.

**We agree that more work is required to better understand how laboratories might overcome the challenges facing them in building networks. The work presented here is a scoping study to help inform discussions about forming a network of NTD laboratories.**

*Where is this indicated in the text?*

I have read this submission. I believe that I have an appropriate level of expertise to confirm that it is of an acceptable scientific standard, however I have significant reservations, as outlined above.

# Peer review: Reviewer response for version 2

**DOI:** 10.5256/f1000research.18267.r39763

**Published:** 2018-11-01

**Authors:** Linda Oskam

**Affiliations:** 1 DATOS B.V., Hoofddorp, The Netherlands

The manuscript makes a first inventory of potential NTD reference laboratories.

 As indicated by the first referee, it highlights the lack of NTD networking capacity. This is not unexpected in the view of this being NEGLECTED tropical disease.

 A number of immediate questions that the authors in my opinion need to address are:

1. The fact that data from the European and African regions are lacking is a major weakness of the article. I would suggest that the authors try to include at least the relevant laboratories from the African region.
2. The authors use a list of 25 NTDs. Which NTDs these are is unclear. WHO uses a list of 20 NTDs ( https://www.who.int/neglected_diseases/diseases/en/). It would be good to indicate which list of NTDs the authors use.
3. Also: NTDs are a heterogeneous lot: parasites, viruses, bacteria, fungi. How realistic is it to assume that dedicated NTD laboratories exist? In most cases it will be one or more departments or groups within bigger laboratories.
4. With regard to accreditation: the laboratories that indicate that they have international accreditation: is the NTD testing portfolio included in their scope of accreditation? Otherwise it does not count as accredited lab.
5. With regard to human resources: are the numbers in the table the total number of staff in the whole lab or the number of staff actually working on NTDs? The latter is the relevant number.
6. Do the authors think that by identifying NTD labs through published literature they may have created a bias towards research-oriented laboratories? Many routine laboratory hardly publish their work (if at all).
7. Introduction, paragraph 4: “The role of the NTD reference laboratories is to maintain their own accreditation…” That is not a role, but a requirement to fully function as a reference laboratory. Supporting others to implement a QMS is a role.
8. Methodology paragraph 5: “Overall 60 countries were identified as being affected by and prioritizing NTDs…” The WHO website (see reference under point 2) puts this number at 149. Please explain the discrepancy.
9. EQA programmes: I am not surprised that not many labs participate in EQA programs as I know from experience that these are often unavailable outside the big diseases. The ISO standard provides a loophole for this in the form of interlaboratory comparisons. Did the authors identify how many labs work with interlaboratory comparisons for their NTD testing?
10. How many of the labs have direct reporting for NTDs to the national disease control programs at MoH level?
11. I am afraid that a network of NTD laboratories is necessary, but will be very difficult to attain: most LMIC have a severe lack of lab staff and a severe lack of funding for their health system in general. NTDs will not be among their first 100 priorities. In many cases NTD testing will have to piggyback on other testing and make use of the leftover resources of the “big” diseases.
  Also, funding for NTD research is limited and haphazard. I think building a sustainable network will be an enormous challenge.
12. I think that ultimately more qualitative research with multiple stakeholders (semi-structured interviews, focus group discussions) are needed to really understand how to best design and implement a sustainable laboratory network for NTDs.

I have read this submission. I believe that I have an appropriate level of expertise to confirm that it is of an acceptable scientific standard, however I have significant reservations, as outlined above.

# Peer review: Reviewer response for version 1

**DOI:** 10.5256/f1000research.17685.r38347

**Published:** 2018-10-10

**Authors:** Charles S. Mgone

**Affiliations:** 1 Hubert Kairuki Memorial University, Dar es Salaam, Tanzania

The manuscript highlights the lack of international, regional and national capacity for reference laboratories and networks for neglected tropical disease diagnosis, research and training. This includes lack of hierarchical referral system that allows escalation of problem solving based on the nature of their complexity and offers supervision and quality assurance along the chain. The paper highlights these problems very well. The data collection was based on laboratory personnel self-reporting after the laboratories were identified by informants and web searching; both inherent with bias. The identification methods used are likely to select well known or well advertised centres while self-reporting is likely to be associated with higher claims of excellence that is real. The lack of information from African and European regions is very glaring and needs to be rectified. Informed willingness of these centres to work as regional reference laboratories needs to be assed, especially after the centres having been made aware of the terms of reference.

I have read this submission. I believe that I have an appropriate level of expertise to confirm that it is of an acceptable scientific standard.

## References

[1] DeanLNjelesaniJMulambaCDacombeRMbabaziPBatesI: Establishing an international laboratory network for neglected tropical diseases: Understanding existing capacity in five WHO regions. *F1000Research*.2018;7: 10.12688/f1000research.16196.1 10.12688/f1000research.16196.1 PMC650995631119028
